# The checkpoint ordering problem

**DOI:** 10.1080/02331934.2017.1341507

**Published:** 2017-10-03

**Authors:** P. Hungerländer

**Affiliations:** ^a^ Massachusetts Institute of Technology, Laboratory for Information & Decision Systems, Cambridge, MA, USA.

**Keywords:** Combinatorial optimization, dynamic programming, integer linear programming, global optimization, facilities planning and design

## Abstract

We suggest a new variant of a row layout problem: Find an ordering of *n* departments with given lengths such that the total weighted sum of their distances to a given checkpoint is minimized. The Checkpoint Ordering Problem (COP) is both of theoretical and practical interest. It has several applications and is conceptually related to some well-studied combinatorial optimization problems, namely the Single-Row Facility Layout Problem, the Linear Ordering Problem and a variant of parallel machine scheduling. In this paper we study the complexity of the (COP) and its special cases. The general version of the (COP) with an arbitrary but fixed number of checkpoints is NP-hard in the weak sense. We propose both a dynamic programming algorithm and an integer linear programming approach for the (COP) . Our computational experiments indicate that the (COP) is hard to solve in practice. While the run time of the dynamic programming algorithm strongly depends on the length of the departments, the integer linear programming approach is able to solve instances with up to 25 departments to optimality.

## 1. Introduction

In this paper we introduce and analyse a new variant of a row layout problem. An instance of the Checkpoint Ordering Problem (COP) consists of *n* one-dimensional departments, with given positive lengths 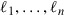 and weights 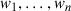, and a checkpoint on a fixed position, e.g. left-aligned or at the center position. The optimization problem can be written down as(1)
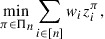



where 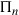 is the set of permutations of the indices 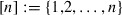 and 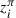 is the distance between the center of department *i* and the checkpoint with respect to a particular permutation 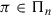.

Let us start with elaborating on the connections of the (COP) to the Linear Ordering Problem (LOP), the Single-Row Facility Layout Problem (SRFLP) and scheduling on identical parallel machines with the objective of minimizing the sum of weighted completion times.

### 1.1. The Single-Row Facility Layout Problem (SRFLP) 

The simplest known layout type is single-row layout. It arises as the problem of ordering stations on a production line, where the material flow is handled by an automated guided vehicle (AGV) in both directions on a straight-line path [1]. An instance of the (SRFLP) consists of *n* one-dimensional departments, with given positive lengths 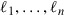, and pairwise weights 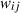. The optimization problem can be written down as(2)
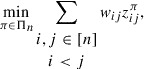



where 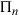 is the set of permutations of the indices [*n*] and 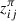 is the center-to-center distance between departments *i* and *j* with respect to a particular permutation 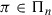. Note that the weights are assumed to be non-negative for all row layout problems to ensure boundedness of the objective value of the optimal layout. For the (SRFLP) 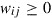 further guarantees that all departments are placed next to each other without spacing. For the (COP) we do not need this restriction on the weights as space between the departments is not allowed.

Several practical applications of the (SRFLP) have been identified in the literature, such as the arrangement of rooms on a corridor in hospitals, supermarkets, or offices [2], the assignment of airplanes to gates in an airport terminal [3], the arrangement of machines in flexible manufacturing systems [1], the arrangement of books on a shelf and the assignment of disk cylinders to files [4].

Similar applications are conceivable for the (COP), e.g. the rooms on a corridor could be arranged such that the weighted sum of their distances with the office of the head is minimized or planes could be assigned to gates such that the weighted sum of their distances from the entrance of the airport terminal is minimized. When comparing the (SRFLP) with the (COP), we observe that the problems are quite similar. One difference is that an (SRFLP) instance has 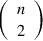 weights while a (COP) instance has only *n* weights.

### 1.2. The Linear Ordering Problem (LOP) 

Ordering problems associate to each ordering (or permutation) of the set [*n*] a profit and the goal is to find an ordering of maximum profit. In the simplest case of the Linear Ordering Problem (LOP) , this profit is determined by those pairs 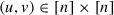, where *u* comes before *v* in the ordering. Thus in its matrix version the (LOP) can be defined as follows. Given an 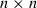 matrix 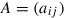 of integers, find a simultaneous permutation 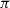 of the rows and columns of *A* such that
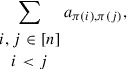



is maximized. Equivalently, we can interpret 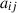 as weights of a complete directed graph *G* with vertex set 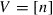. A tournament consists of a subset of the arcs of *G* containing for every pair of nodes *i* and *j* either arc (*i*, *j*) or arc (*j*, *i*), but not both. Then the (LOP) consists of finding an acyclic tournament, i.e. a tournament without directed cycles, of *G* of maximum total edge weight.

Although the (LOP) and the (COP) have apparently a similar structure, it is harder to directly relate these two problems. We will show in Section 2 that the (COP) with left-aligned or right-aligned checkpoint is in fact a (LOP) with some additional structure and can be solved efficiently by a greedy algorithm.

### 1.3. Scheduling on identical parallel machines

Furthermore we can relate the (COP) to the NP-hard [5] scheduling on identical parallel machines with the objective of minimizing the total weighted completion time that is defined as follows: We are given a set of jobs 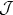 that have to be scheduled on *m* identical parallel machines. Each job 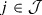 is specified by its processing time 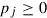 and by its weight 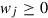.

Every machine can processat most one job at a time, and every job has to be processed on one machine in an uninterrupted fashion. The completion time of job *j* is denoted by 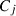. The goal is to minimize the total weighted completion time 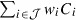. In the standard classification scheme of [6], this scheduling problem is denoted by 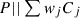 for *m* part of the input, and by 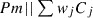 for constant *m*.

For the special case of only one machine the problem can be solved in polynomial time by Smith’s Rule [7] that suggests to process the jobs in the order of non-increasing ratios 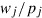. For a constant number 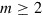 of machines, the problem is weakly NP-hard as it can be solved in pseudopolynomial time by dynamic programming approaches [8–10]. For *m* part of the input, the problem is NP-hard in the strong sense by transformation from 3-PARTITION [11]. For further details on the complexity of various related variants of this scheduling problem we refer to problem SS13 in [11]. For a more general overview on machine scheduling we refer to the survey article by [12].

From a computational point of view 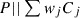 has been tackled by various branch-and-bound methods [13–17], for which determining the optimal solution of instances with 30 or more jobs and two or more identical machines is typically difficult [18].

When comparing 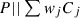 and the (COP), we observe that there two important differences between the two problems:

(1) The checkpoint must not lie exactly at a splitting point of two departments but it can also be covered by a department. I.e. the checkpoint does not necessarily define a partition of the departments. When considering a scheduling set-up the (COP) can be described as follows: It is allowed to split one arbitrary job into two parts at any point and then the two parts have to be scheduled first on the two machines.
(2) For the (COP) the sum of the lengths of the departments that are placed to the left and to the right of the checkpoint are predetermined through the position of the checkpoint. E.g. for a centered checkpoint the sum of the lengths of the departments to the left and to the right of the checkpoint has to be equal. For 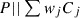 the identical machines typically have no capacity restrictions.

Due to these differences it is not possible to directly carry over polyhedral results, dynamic programming algorithms and (mixed) integer linear programming (ILP) models and their corresponding approximation results [19] from scheduling on identical parallel machines to the (COP).

### 1.4. Toy examples

**Figure 1.**
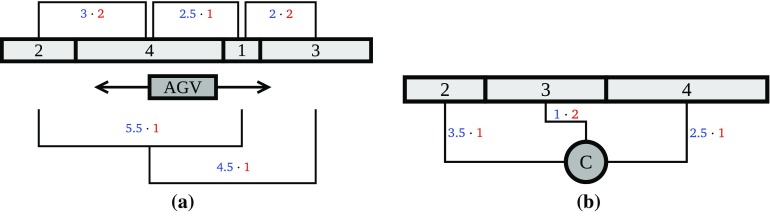
We are given the following data: 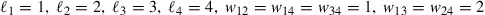. In (a) we display the optimal layout for the (SRFLP) with corresponding costs of 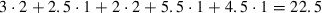. And in (b) we depict the optimal layout for the (COP) with department 1 assigned to row 2 and all other departments assigned to row 1, disregarding the weights 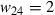 and 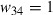. Further we assume that the checkpoint lies at the center. Then the costs of the optimal (COP) layout are 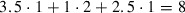.

Now let us further clarify the similarities and differences of the (SRFLP) and the (COP) with the help of a toy example: We consider 4 departments with lengths 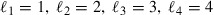. Additionally we are given the pairwise weights 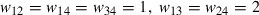. For the (COP) we assign department 1 to row 2 and all other departments to row 1 and hence disregard the weights 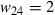 and 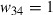. Figure 1 illustrates the optimal layouts and the associated costs for both problems.

**Figure 2.**
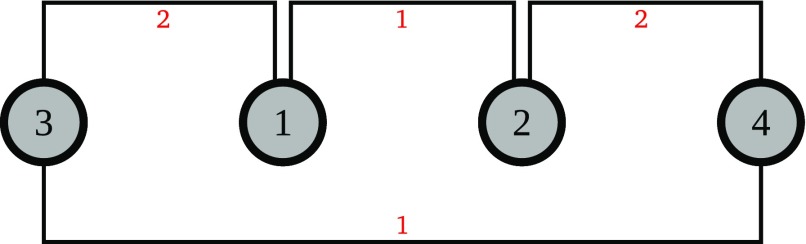
We are given 4 objects and the weights 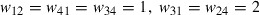. We display the optimal (LOP) solution with the corresponding benefit of 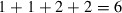.

Finally we also want to clarify the workings of the (LOP) with the help of a toy example. We consider 4 objects and the weights 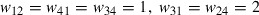. Figure 2 illustrates the optimal ordering of the objects and the corresponding benefit.

### 1.5. Outline

The main contributions of this paper are the following:

- We propose a new combinatorial optimization problem that is both of theoretical and practical interest.
- We study the complexity of the (COP) and its special cases, pointing out several connections to related problems.
- We propose two exact approaches for the (COP), namely a dynamic programming algorithm and an ILP approach.
- We demonstrate the practical difficulty of the (COP) in a computational study. In this context let us also refer to our companion paper [20] for a comparison of the empirical difficulty of several row facility layout problems (including the (COP) ) on a variety of well-known benchmark instances.

The paper is structured as follows. In Section 2 we study the complexity of the (COP) and its special cases. In Sections 3 and 4 we suggest a dynamic programming algorithm and an ILP approach for the (COP) respectively. Finally in Section 5 we conduct computational experiments, indicating the practical applicability and limitations of the approaches suggested. Section 6 concludes the paper.

## 2. Complexity of the Checkpoint Ordering Problem

Consider the decision variant of the (COP) : given some value *M* we ask whether there exists a permutation of the departments such that the obtained costs are at most *M*:

**Decision Checkpoint Ordering (DCO):**

*Instance*: *n* departments with given lengths 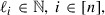 and integer weights 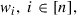 and a checkpoint on a fixed position.

*Question*: Is there an ordering 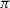 of the departments such that the total costs 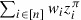 are 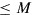
?

In the following proof we assume w.l.o.g. that the checkpoint is located at the center in order to simplify the presentation.

Theorem 1: DCO is NP-complete.

**Proof 1**It is clear that DCO 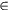 NP since a nondeterministic algorithm needs only to guess an ordering 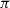 and then can check in polynomial time if the corresponding costs are 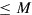.

To prove that DCO is NP-complete, we give an NP-complete problem and a polynomial-time transformation to DCO. The following problem is NP-complete (see Section 3.1.5 of [11], originally proven by [21]):

**PARTITION:**

*Instance*: A finite set *A* and a ‘size’ 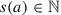 for each 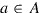 such that 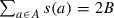 is even.

*Question*: Is there a subset 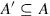 such that 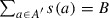
?

Now we transform an instance of PARTITION to an instance of DCO as follows. We replace each element 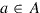 with given size *s*(*a*) by a department *a* with length 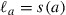 and weight 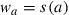. Additionally to this local replacement we use an enforcer by introducing a further department *t* with length 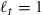 and weight 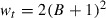. Clearly this DCO instance can be constructed from the PARTITION instance in polynomial time.

If the ordering 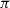 is optimal then the center of department *t* is located exactly above the checkpoint because of the large weight of department *t*. Due to the definition of the weights of the departments 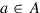, it does not change the objective value if we switch the positions of two departments both located left or right of *t*. Hence the only way to influence the objective value is to decide whether the departments should be located left or right of *t*. If we can find a subset 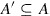 of the departments such that 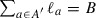 and place them left of *t* and all other departments right of *t*, then the corresponding ordering 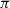 is for sure optimal. If the sum of lengths of the departments left of *t* in the optimal ordering 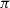 does not give *B*, then there exists no subset 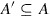 such that 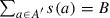.

In summary we have shown that there exists an ordering 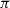 of the departments such that the total costs are 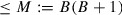 if and only if there exists a subset 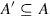 of the corresponding PARTITION instance such that 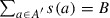.

In the layout context the above result can be interpreted as follows: The minimization of the inter-row costs that occur in all multi-row layout problems is NP-hard, even in its simplest version. This is not only an interesting theoretical insight on its own but also supports our definition of the Single-Row bound in our companion paper [20]. Next let us consider some more specialized versions of the (COP) that turn out to be solvable by a greedy algorithm and hence in particular in polynomial time.

**Figure 3.**
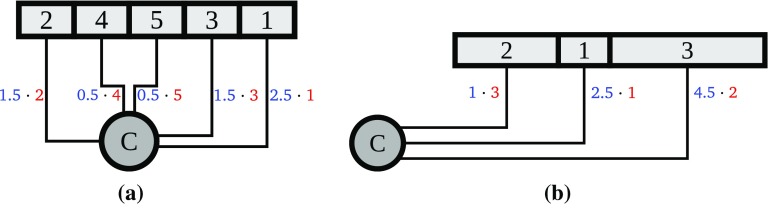
In (a) we display the optimal layout for the (COP) with identical department lengths equal to 1 and weights 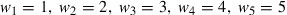. The corresponding layout costs are 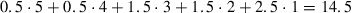. In (b) we show the optimal layout for the (COP) with left-aligned checkpoint on the following instance with 3 departments: 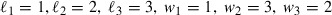. The associated layout costs are 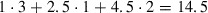.

First we assume that all departments have the same length. In this case the optimal permutation of the departments can be obtained by a simple greedy selection. We choose the permutation 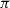 with the following property: The higher the weight of a department the smaller is its distance from the checkpoint. Next we give a short formal argument for the above claim and refer to Figure 3 for a toy example of this variant of the (COP).

Fact 2: A permutation 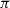 is optimal for the (COP) with identical department lengths iff it ensures the inequalities(3)
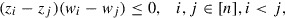

where 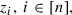 denotes the distance of the center of department *i* from the checkpoint.

**Proof 2**The change of the objective function caused by swapping two departments *i* and *j* is 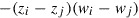. Hence in particular the change in the objective function caused by this swap is independent of the length and weights of all other departments 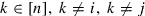. Now assume that there exists an optimal permutation that does not ensure one inequality in (3), i.e. 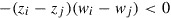 for some departments *i* and *j*. Now swapping the two departments improves the objective value. As all other departments are not affected by pairwise swaps, it is not possible to improve the objective value by an arbitrary number of pairwise changes if (3) holds. Hence (3) is not only a necessary condition but also a sufficient one.

Note that the special case of the (SRFLP) where all department lengths are equal and the weights are binary is still NP-hard [22]. This problem is called Minimum Linear Arrangement (LA), belongs to the class of graph layout problems and is NP-hard even if the underlying graph *G* is bipartite [11]. (LA) was originally proposed by Harper [23,24] to develop error-correcting codes with minimal average absolute errors and was since then applied to VLSI design [25], single machine job scheduling [26,27] and computational biology [28,29]. There exist approximation algorithms for (LA) with performance guarantee 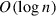 [30,31] and 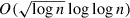 [32,33]. For further details on graph layout problems we refer to the survey paper of Díaz et al. [34].

Next we assume that the checkpoint is left-aligned or right-aligned. Also in this case the optimal permutation of the departments can be obtained by a simple greedy selection. We choose the permutation 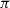 with the following property: The higher the relative weight 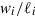 of a department *i*, the smaller is its distance from the checkpoint. Next we give a short formal argument for the above claim and refer to Figure 3 for a toy example of this variant of the (COP) . To facilitate the presentation of the proof, we assume w.l.o.g. that

(1) the checkpoint is left-aligned and
(2) the relative weights 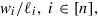 are all distinct.

The following result is in fact known as Smith’s Rule [7] in the scheduling context, where it describes a greedy algorithm to solve single-machine scheduling with the objective of minimizing the sum of completion times. For convenience we restate the proof in our notation.

Fact 3: [7] The permutation 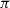 is optimal for the (COP) with a left-aligned checkpoint iff it satisfies the conditions(4)
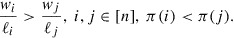

**Proof 3**Assume that there exists an optimal permutation that does not satisfy condition (4) for two departments *i* and *j*: 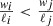 and 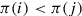. Then there are also two neighboring departments *k* and *l* (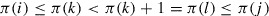) that do not satisfy condition (4): 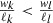 and 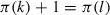. Now if we swap *k* and *l*, the value of the objective function changes by the term 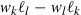. But this term is negative as 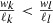 holds which yields a contradiction to the assumption that the permutation was optimal. Finally note that condition (4) defines a unique permutation, hence it is not only necessary but also sufficient.

We can interpret the (COP) with left-aligned checkpoint also as a (LOP) with special structure. We collect the lengths of the departments in a column vector 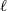 and the weights of the departments in a column vector *c*. Now we aim to find a simultaneous permutation 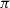 of the rows and columns of 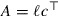 such that
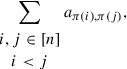



is minimized. Contrary to the general (LOP), which is NP-hard [22], the matrix entries of *W* are not independent but determined by an outer product of two vectors. We have just seen in Fact 3 that the (COP) with left-aligned checkpoint can be solved by a simple greedy heuristic. Hence this special structure of the (LOP) cost matrix *W* as an outer product of two vectors is the reason why this (LOP) version can be solved in polynomial time.

We summarize the above results as follows: The (COP) is NP-hard and its ‘hard’ part is to determine where the centers of the departments are located with respect to the checkpoint in the optimal solution. In the following section we show that the (COP) with one checkpoint that is neither left- nor right-aligned can be solved by a dynamic programming algorithm and hence is NP-hard in the weak sense. If the number of checkpoints is not part of the input, then the (COP) is NP-hard in the strong sense.

## 3. A Dynamic Programming Algorithm for the (COP) 

We exploit the following two simple properties of (COP) layouts for designing a dynamic programming algorithm:

- In every (COP) layout there is at most one department covering the checkpoint. All other departments lie completely to the left or to the right of the checkpoint.
- In an optimal (COP) layout all departments, except for the department covering the checkpoint, have to be arranged in non-increasing order from the checkpoint to the border of the layout with respect to their relative weights 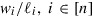. This structural characteristic is denoted as V-shaped property [35,36].

In order to simplify the description of our dynamic programming algorithm we assume w.l.o.g. that the checkpoint is centered and hence that the sum of the lengths of the departments to the left and to right of the checkpoints has to be equal. Now let us outline the workings of our dynamic programming algorithm in detail.

The input for our algorithm is a (COP) instance, where the list of departments is given in non-decreasing order with respect to their relative weights 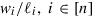. First we choose one of the given departments as center department that covers the checkpoint, where 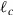 is the length of the center department, 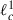 is the length of the part of the center department left to the checkpoint and 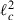 is the length of the part of the center department right to the checkpoint. Hence 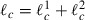 and additionally due to symmetry we can always assume w.l.o.g. that 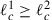 holds.

Next we choose an alignment of the center department above the checkpoint: We start with the right end of the center department above the checkpoint and then shift the center department 0.5 to the right in each iteration until the center of the center department is placed directly above the checkpoint. Clearly it suffices to consider 0.5 shifts of the center department as the checkpoint is centered and 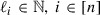. In the inner loops we determine with the help of the following recursive relation whether to place the remaining departments to the left or to the right of the checkpoint(5)
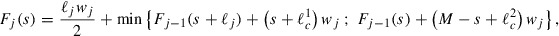



where *s* indicates the remaining free space to the left of the checkpoint and *M* gives the overall remaining free space either to the left or to the right of the checkpoint, which is equal to the sum of the lengths of the departments not yet assigned. As we arrange the departments in non-increasing order with respect to their relative weights 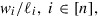 from the checkpoint to the border, the V-shaped property of the resulting layout is ensured.

We refer to Algorithm 1 for a detailed description of our dynamic programming algorithm that we implemented in C. In Section 5 we computationally compare the dynamic programming algorithm to an ILP approach for the (COP) that we suggest in the following section. In summary our dynamic programming algorithm for solving the (COP) runs in pseudo-polynomial time, to be precise in 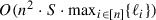 with(6)
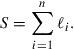



Hence the (COP) with one checkpoint is NP-hard in the weak sense. If the number of checkpoints is not part of the input, then the (COP) is NP-hard in the strong sense, which can be proven by a deduction from 3-PARTITION.

**Figure 4.**
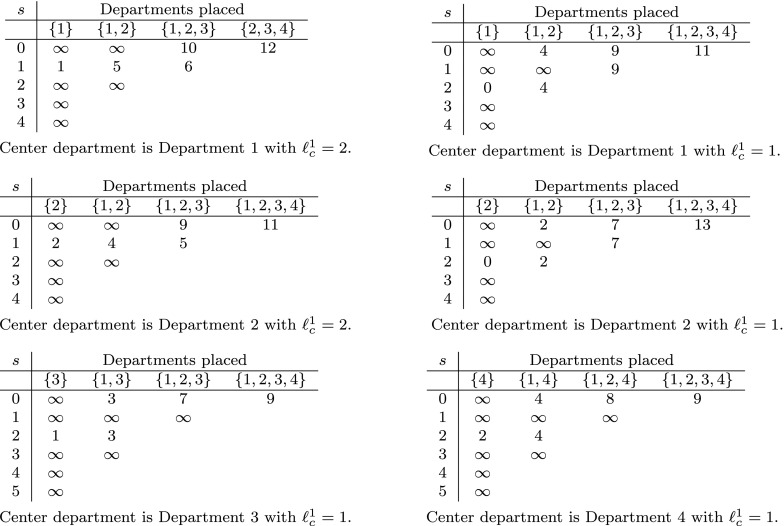
All 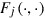-values for 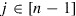 that were determined by our dynamic programming algorithm for a toy example with centered checkpoint and input data 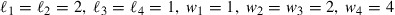. The optimal objective value is 9 and the optimal layouts are (1, 3, 4, 2), (1, 4, 3, 2), (2, 3, 4, 1) and (2, 4, 3, 1).

To further clarify the workings of our dynamic programming algorithm let us consider a toy example with centered checkpoint and the following input data: 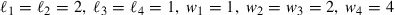. The optimal objective value is 9 and the optimal layouts are (1, 3, 4, 2), (1, 4, 3, 2), (2, 3, 4, 1) and (2, 4, 3, 1). All 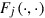-values for 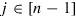 that were determined by our algorithm for this example are stated in Figure 4.


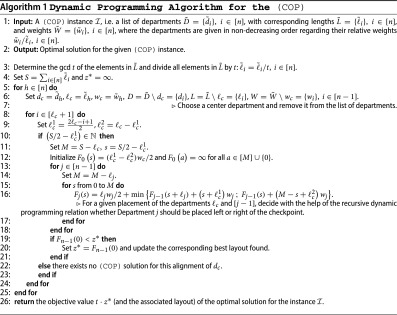


## 4. An ILP Formulation for the (COP) 

In the section we propose an ILP approach for solving the (COP) . To simplify the notation, we consider the (COP) on 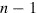 departments. First we introduce binary ordering variables 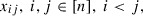 with the interpretation(7)
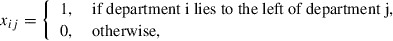



in order to relate the positions of the 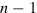 departments to each other and to the checkpoint *n* that is again w.l.o.g. assumed to be centered. To ensure transitivity on the ordering variables we use the 3-cycle inequalities(8)
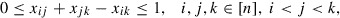



that rule out the existence of directed 3-cycles and are sufficient for guaranteeing that there is no directed cycle.

Now the distances of the departments from the checkpoint can be expressed as quadratic terms in ordering variables: For department 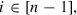 we sum up the lengths of the departments left of *i* plus 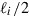 and denote it by 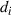. Furthermore we compute the position of the checkpoint 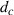 as *S* / 2, i.e. the total length of the departments divided by 2. Then we subtract 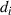 from 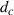. This difference gives the distance of the center of department *i* from the checkpoint, if department *i* is located to the left of the checkpoint. If department *i* is located to the right of the checkpoint, this difference is minus the distance of the center of department *i* from the checkpoint. Therefore we multiply this difference by the term 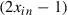 that is 1, if the center of department *i* lies to the left of the checkpoint and 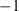 if the center of department *i* lies to the right of the checkpoint:(9)
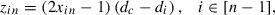



with
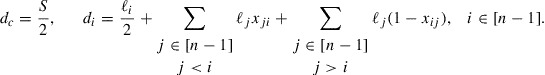



The additional multiplication with 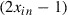 ensures a correct calculation of all distances through the following constraints:(10)
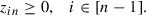



Expanding and simplifying (9) yields(11)
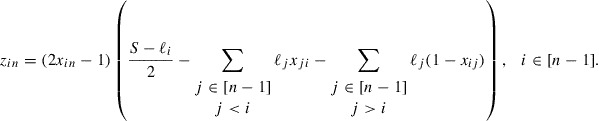



To model the (COP) as an ILP we apply standard linearization and introduce new variables for all products of ordering variables in (11):(12)
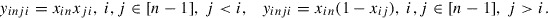



Note that we have to introduce 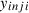 for all 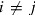 as the variable *i* appears twice in the indices. Now (11) can be further rewritten as:(13)
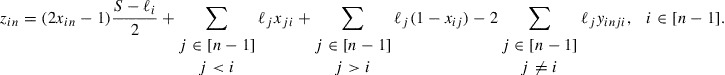



Moreover we use the following standard constraints to relate the orderings variables and their products:(14)
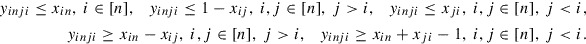



In summary we obtain the following ILP model for the (COP) :
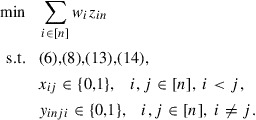



In the following section we computationally compare an ILP approach based on this model with our dynamic programming algorithm introduced in the previous section.

## 5. Computational experiments

We report the results of computational experiments with our dynamic programming algorithm and ILP approach respectively. All computations were conducted on an Intel Xeon E5160 (Dual-Core) with 2 GB RAM, running Debian 5.0 in 64-bit mode. The algorithms were implemented in C (dynamic programming algorithm) and Gurobi 6.5 (ILP) respectively. To generate (COP) instances we use layout benchmark instances from the literature by simply choosing one department *i* as checkpoint and deducing the (COP) weights from the pairwise weights as follows: 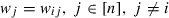. In order to test the effect of varying department lengths on our approaches, we generated additional instances using the same weights but substituting the original department lengths lying in the range of 1–20 with random department lengths between 1 and 10,000. We add an *L* to the instance name for indicating the instances with the new random department lengths. All instances considered can be downloaded from http://tinyurl.com/layoutlib.

In Tables 1 and 2 we state the results of our ILP approach. For instances with up to 25 departments the ILP succeeds in determining the optimal solution. For instances with a higher number of departments (except from ‘ste36.5’) we observe quite large gaps even after 24h. The performance of the ILP approach is hardly influenced by the department lengths as can be seen by comparing the results in Tables 1 and 2.

**Table 1.** Results obtained by our ILP approach on instances with regular department lengths using Gurobi 6.5 [37] restricted to one thread on our machine with a time limit of 24 h. The running times are given in sec or in h:min:sec respectively.

Instance	Lower bound	Upper bound	Gap in %	Time	B&B nodes
P15	189	189	0.0	30	15360
P17	675.5	675.5	0.0	1:59:00	2368737
P18	679.5	679.5	0.0	3:05:00	3241655
H_20	710	710	0.0	5:30:00	4767725
N25_05	368	368	0.0	17:00:00	3885648
H_30	645	1441	55.2	24:00:00	1593134
N30_05	973.5	3224.5	68.8	24:00:00	1514491
Am33_03	600.5	1898.5	86.4	24:00:00	106547
Am35_03	185.5	2132.5	91.3	24:00:00	210111
ste36.5	1444	1444	0.0	1:24:00	38781
N40_5	81	2772	97.1	24:00:00	42135
sko42-5	84	4059	97.9	24:00:00	27352

In Tables 3 and 4 we state the results of our dynamic programming algorithm that determines the optimal solution for all benchmark instances with short department lengths within seconds. As our dynamic programming algorithm runs in 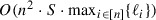, it is much slower on the benchmark instances with large department lengths, where it is able to solve instances with up to 33 departments. If the dynamic programming algorithm does not finish within the time limit, then it does not provide a lower bound and hence also no global gap for the optimal solution.

**Table 2.** Results obtained by our ILP approach on instances with large department lengths using Gurobi 6.5 [37] restricted to one thread on our machine with a time limit of 24 h. The running times are given in min:sec or in h:min:sec respectively.

Instance	Lower bound	Upper bound	Gap in %	Time	B&B nodes
P15L	148059.5	148059.5	0.0	1:08	24589
P17L	438935.5	438935.5	0.0	28:25	504935
P18L	589182.5	589182.5	0.0	24:51	225180
H_20L	245666	245666	0.0	2:43:41	1109189
N25_05L	401212.5	555504.5	27.7	24:00:00	3050435
H_30L	389374	754755	48.4	24:00:00	4860433
N30_05L	239436	935762.5	74.4	24:00:00	483913
Am33_03L	138929.5	1094984.5	87.3	24:00:00	322711
Am35_03L	24.5	1645539.5	100	24:00:00	44175
ste36.5L	813222.5	814001.5	0.1	24:00:00	662589
N40_5L	192379	2518555	92.4	24:00:00	535358
sko42-5L	67631	2363015	97.1	24:00:00	411416

**Table 3.** Results obtained by our dynamic programming algorithm on instances with regular department lenghts. The running times are given in sec.

Instance	Optimum	Time
P15	189	0.01
P17	675.5	0.02
P18	679.5	0.02
H_20	710	0.15
N25_05	368	0.03
H_30	1439	0.46
N30_05	3191.5	3.70
Am33_03	1879.5	0.55
Am35_03	2116.5	0.32
ste36.5	1444	1.73
N40_5	2747	0.64
sko42-5	3694	6.42

**Table 4.** Results obtained by our dynamic programming algorithm on instances with large department lengths. The running times are given in min:sec or in h:min:sec respectively. The entries * indicate that the algorithm did not finish within the time limit of 24 h.

Instance	Optimum	Time
P15L	148059.5	47:45
P17L	438935.5	1:54:44
P18L	589182.5	3:58:42
H_20L	245666	1:32:45
N25_05L	554740.5	10:31:00
H_30L	752966	9:58:20
N30_05L	920622.5	16:10:33
Am33_03L	1078710.5	20:51:16
Am35_03L	*	*
ste36.5L	*	*
N40_5L	*	*
sko42-5L	*	*

Comparing our two approaches we observe that the dynamic programming algorithm is clearly superior for all benchmark instances from the facility layout literature that only contain departments with short lengths 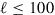. For instances with department lengths up to 10000 the dynamic programming algorithm is still preferable although there already exist instances (see e.g. ‘ste36.5’) on which the ILP yields clearly better results. Finally for instances with even larger department lengths the ILP gradually outperforms the dynamic programming algorithm.

## 6. Conclusion

In this paper we proposed a new variant of a row facility layout problem and two exact algorithms for solving it. The Checkpoint Ordering Problem (COP) is weakly NP-hard. It is both of theoretical and practical interest and has several important relations to other well-studied combinatorial optimization problems. In our computational study we showed that the (COP) is hard to solve in practice for both dynamic programming and ILP approaches.

It would be interesting to examine if the models, results and algorithms for scheduling on two parallel machines, which is a very well-studied problem, can be used to obtain stronger approximation results and/or to design stronger exact approaches for the (COP) in particular and layout problems in general.
